# Four-year follow-up study in a NF1 Boy with a focal pontine hamartoma

**DOI:** 10.1186/1824-7288-39-10

**Published:** 2013-02-11

**Authors:** Pasquale Parisi, Severino Persechino, Maria Chiara Paolino, Francesco Nicita, Isabella Torrente, Alessandro Bozzao, Maria Pia Villa

**Affiliations:** 1 Child Neurology, Chair of Paediatrics, NESMOS Department, Faculty of Medicine and Psychology, Sapienza University c/o St Andrea Hospital, Via di Grottarossa 1035-1039, 00189, Rome, Italy; 2 NESMOS Department, Faculty of Medicine and Psychology, Sapienza University, Rome, Italy; 3 Faculty of Medicine “Sapienza” University c/o Umberto I Hospital, 00161, Rome, Italy; 4 IRCCS-CSS San Giovanni Rotondo and CSS-Mendel Institute, 00189, Rome, Italy; 5 Chair of Neuroradiology, NESMOS Department, Faculty of Medicine and Psychology, Sapienza University, Rome, Italy

**Keywords:** Brainstem tumor, Diffusion weighted imaging, Intra-familial phenotype variability, MR-spectroscopy, Neurofibromatosis, Pontine hamartoma

## Abstract

Neurofibromatosis is a collective name for a group of genetic conditions in which benign tumours affect the nervous system. Type 1 is caused by a genetic mutation in the NF1 gene (OMIM 613113) and symptoms can vary dramatically between individuals, even within the same family. Some people have very mild skin changes, whereas others suffer severe medical complications. The condition usually appears in childhood and is diagnosed if two of the following are present: six or more café-au-lait patches larger than 1.5 cm in diameter, axillary or groin freckling, 2 or more Lisch nodules (small pigmented areas in the iris of the eye), 2 or more neurofibromas, optic pathway gliomas, bone dysplasia, and a first-degree family relative with Neurofibromatosis type 1. The pattern of inheritance is autosomal dominant, however, half of all NF1 cases are ‘sporadic’ and there is no family history. Neurofibromatosis type 1 is an extremely variable condition whose morbidity and mortality is largely dictated by the occurrence of the many complications that may involve any of the body systems. We describe a family affected by NF1 in whom genetic molecular analysis identified the same mutation in the son and father. Routine MRI showed pontine focal lesions in the eight-year-old son, though not in the father. We performed a four years follow-up study and at follow-up pontine hamartoma size remained unchanged in the son, and the father showed still no brain lesions, confirming thus an intra-familial phenotype variability.

## Background

Neurofibromatosis type 1 (NF1) (OMIM#162200) [1] has a prevalence of 1 in 4,000 individuals in the general population. It is an extremely variable condition whose morbidity and mortality is largely dictated by the occurrence of the many complications that may involve any of the body systems [2]. CNS tumours may lead to major morbidity and mortality, despite the fact that most are grade I pilocytic astrocytomas [3]. Optic pathway [4] and brainstem [5] gliomas are the prevalent CNS tumours in NF1. Studies in the literature, most of which are based on children, have shown that these tumours are less aggressive than their counterparts in non-NF1 patients [5]. In particular, as a rule, brainstem gliomas behave much less aggressively in NF1 patients than in other patients. Hamartomas or focal areas of high signal intensity can be seen in ≤ 93% of patients with NF-1 [6]. They tend to show T2 hyperintensity but no mass effect, edema, or contrast enhancement. The brain stem, cerebellar peduncles, and basal ganglia are the most preferred locations. The exact nature of hamartomas is not clear. Although the morphologic criteria are often diagnostic in making a differentiation between hamartomas and brain stem glioma, some cases can present diagnostic difficulties and MRspectroscopy can provide additional information. Zamboni et al. detected using diffusion tensor imaging, globally elevated fractional anisotropy (FA) and decreased apparent diffusion coefficient (ADC) values in the mature brains of patients with NF-1, which is most likely a consequence of diffuse and basic alterations in cerebral microstructure that result from the underlying gene mutation. Diffusion changes might be a more sensitive marker for progression of the disease than conventional imaging findings [7].

We describe a family affected by NF1 in whom genetic molecular analysis identified the same mutation in the son and father. Routine MRI showed pontine focal lesions in the eight-year-old son, though not in the father. Interestingly, an isolated focal pontine localization is exceptionally reported in brainstem lesions NF1 associated in pediatric age [3-5]. MR spectroscopy (MRS) imaging and diffusion-weighted imaging (DWI) were also performed in both father and son. At follow-up, four years later pontine hamartoma size remained unchanged in the son, and the father showed still no brain lesions, confirming thus an intra-familial phenotype variability.

## Case presentation

**Figure 1:**
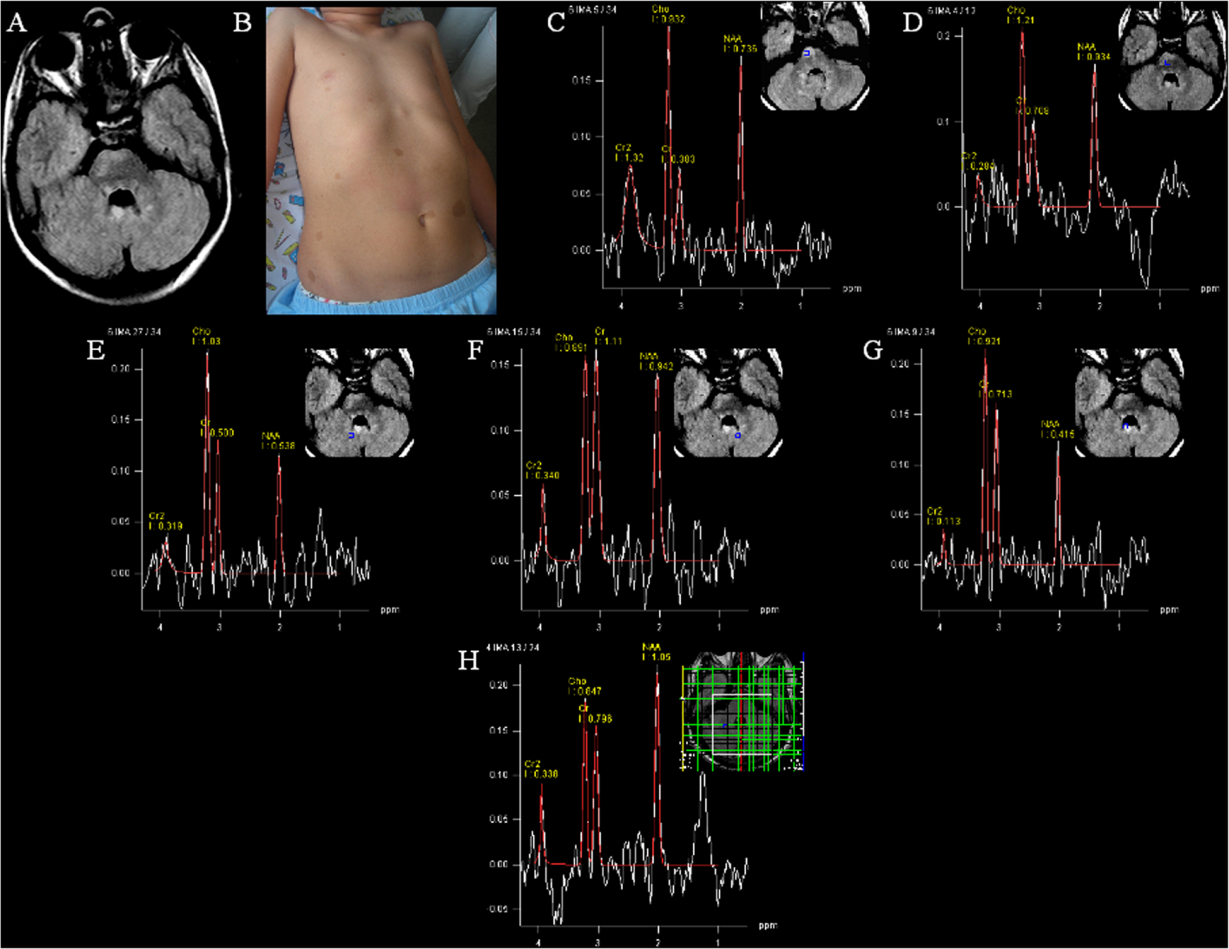
**Neuroradiological and clinical evaluation performed at admission in the child and his father.** MRS positioned on the brainstem and subcortical cerebellar white matter on the basis of FLAIR signal alteration images (**A**); presence of numerous cafe´ au lait patches in the child (**B**); MRS documented increased Cho/Cr resonance intensities in the voxels located inside and at the border of the MRI signal alterations in the brainstem (inside mean value Cho/Cr: 2.43; at the border Cho/Cr: 1.70 (**C**, **D**), in normal appearing white matter between the cerebellar hemispheres (right side Cho/Cr: 2.06 (**E**) (left side Cho/Cr: 0.80 (**F**). MRSI voxels at the level of focal cerebellar lesion documented Cho/Cr: 1.29 (**G**). Nor brain lesions neither metabolic changes in the father’s MRS (**H**).

An 8-year-old boy was referred to our Child Neurology Outpatient Service because of “headache” attacks with “migraine without aura” (MoA) features, according to the International Classification of Headache Disorders. Although the boy’s father was affected by Neurofibromatosis type I, diagnosed according to the international criteria, he had always refused to undergo the molecular genetic analysis for confirmation of the clinical diagnosis; the boy’s late paternal grandfather was also reported to have been clinically affected by NF1. The boy’s birth was uneventful and his neurological and psychomotor development were normal, apart from a slight speech delay. Weight at birth was 3.200 kg, head circumference 35 cm and length 50 cm. There was no family history of epilepsy or migraine, nor personal history of benign paroxysmal childhood vertigo. His 14-year-old brother and his mother were normal at the physical examination. Several EEGs performed between three and eight years of age were reported to be normal. The paediatric, neurological and ophthalmologic examinations confirmed the clinical diagnosis of NF1, with the presence of numerous cafe´ au lait patches (Figure 1B) with axillary freckling and five Lisch nodules (iris hamartomas) seen at the slit-lamp examination. Moreover, the neurological examination showed cranial nerve impairment from the 9^th^ to 12^th^ cranial nerves and slight right upper arm impairment at the Mingazzini item. In view of the strongly suspected diagnosis of NF1 and the afore-mentioned neurological impairment, conventional as well as diffusion tensor and multivoxel spectroscopy MR investigations in both the father and son were planned. The MRI examination was performed repeated at the 6-month and the fourth-years follow-up visit.

The father’s MRI study excluded brain focal lesions and metabolic changes, whereas the son’s MRI study revealed cerebral lesions (Figure 1A), with focal abnormalities on T2-weighted images in the basal ganglia, brainstem, cerebral and cerebellar regions.

The ADC and FA values of the cerebral and cerebellar apparent normal white matter were not significantly different compared with those of 10 pediatric normal controls, (recruited from our outpatient pediatric surgery, with no reported neurological disease, after parents provided written informed consent to the study), so the results are not here reported.

On the contrary, the MRS revealed differences between the brainstem voxel lesion and the border as well as different cerebellar hemispheres values; increased Cho/Cr resonance intensities in the voxels located inside and at the border of the MRI signal alterations in the brainstem (inside mean value Cho/Cr: 2.43; at the border Cho/Cr: 1.70 (Figure 1C, D ), significantly different values in normal appearing white matter between the cerebellar hemispheres (right side Cho/Cr: 2.06 (Figure 1E) (left side Cho/Cr: 0.80 (Figure 1F). MRSI voxels obtained at focal cerebellar lesion documented Cho/Cr 1.29 (Figure 1G). There were no metabolic changes in the father’s MRSI (Figure 1H).

To confirm the clinical NF1 diagnosis, molecular genetic analysis was performed at the Mendel Institute in Rome. After informed consent was obtained DNA from peripheral blood was extracted from each patient (son and father) by means of one of several standard procedures. The identification of NF1 gene mutations was carried out using dHPLC on a 3500HT WAVE DNA fragment analysis system (Transgenomic, Crewe, UK) equipped with a DNASep column (Transgenomic, Crewe, UK). PCR products were examined for heteroduplexes with a separation flow rate of 1.5 ml/min, through a 5% linear acetonitrile gradient. The first base (position +1) of the initiator methionine is taken as the start of the cDNA. dHPLC and sequencing analyses detected the mutation c.586 + 1G > A in intron 4b of the *NF1* gene. The presence of this mutation was confirmed in our patient’s father [8,9].

**Figure 2:**
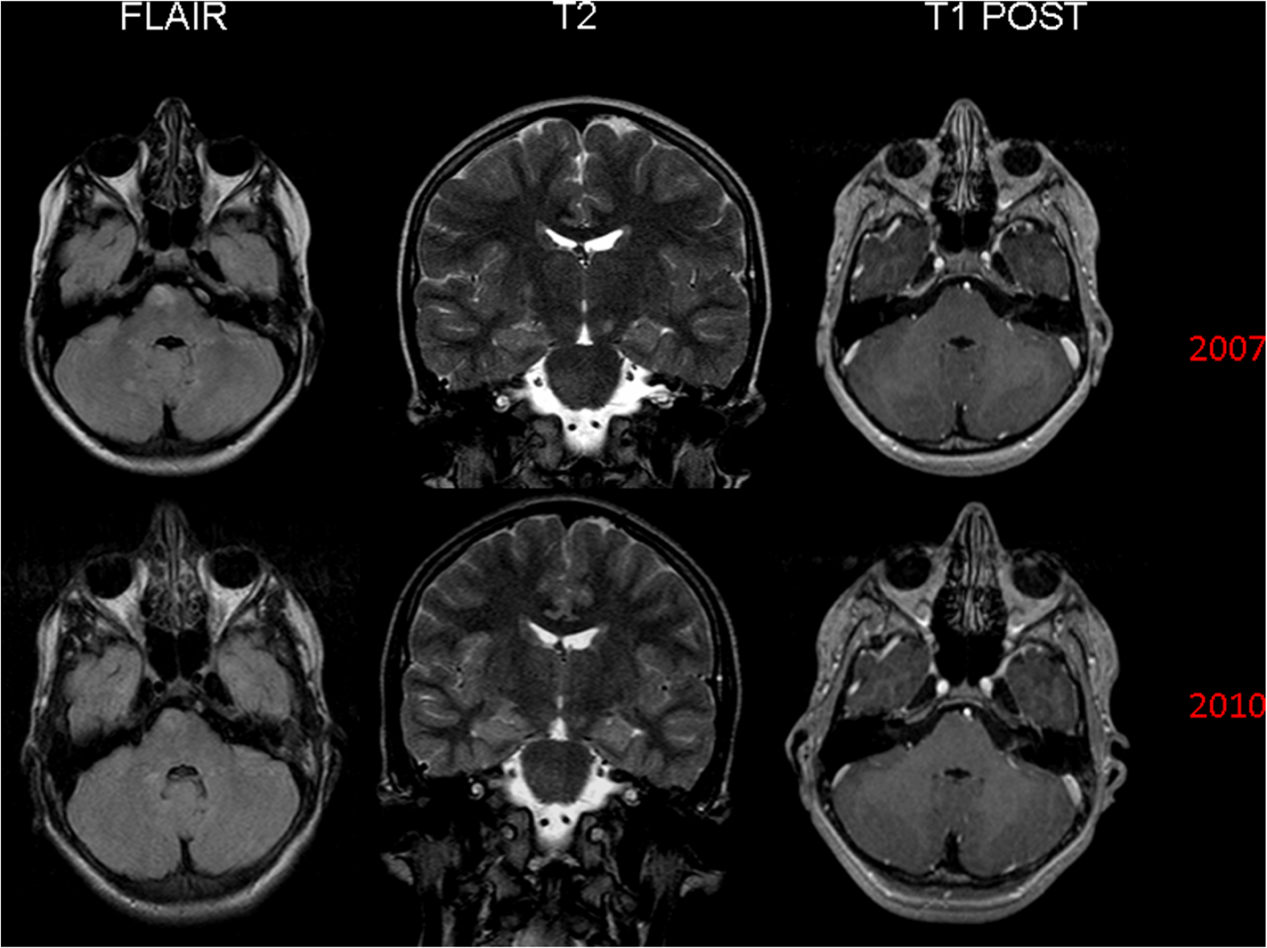
**MRI (Flair, T2, and T1 sequences) performed in the child at the admission (2007) and four years later (2010). 2007:** Hamartoma in the right pontine region and swallen aspect of the omolateral cortico-spinal tract. High signal intensity foci diffuse in the cerebellar emispheres (T2 hyperintensities). **2010**: unmodified size of the pontine hamartoma and persistent bulge of the right cortico-spinal tract. Unchanged the UBOs lesions too.

Ten months after having come to our paediatric headache centre, the patient’s clinical picture has substantially remained the same as well as after four years of follow-up. MRI showed unchanged the size of pontine hamartoma at four years of follow-up (Figure 2: 2007 and 2010).

## Conclusions

The family we describe confirms that a father and son with the same NF1 genetic defects may not present the same clinical picture, confirming thus the possible occurrence of an intra-familial variability [1,2].

To date, contrasting literature data have been published on the usefulness and early predictive value of both DWI and MRS [7,10-14]. Eastwood et al., investigating the brain of children with NF1 by means of DWI, reported myelin disorder, such as reduced amount of myelin and increased myelin turnover or demyelination [11]. No significant differences in metabolites in the normal appearing brain in NF1 patients (either with or without focal lesions) when compared with healthy volunteers were reported [12], while others [13] reported abnormal metabolic changes in the corresponding area of normal appearing brain at MRI in a patient who had a hamartoma at an MRI examination performed one year before. Alkan A et al. described in a total of 30 NF1 patients, higher ADC values both in “T2 hyperintensities” and in the normal appearing locations as hippocampus and thalamus in the patients with NF1. They showed that the detection of lesions might be independent of MRI appearance in NF1, i.e. although the brain is affected, MRI appearance may be normal. Therefore, they suggested that DWI and ADC values should also be utilized in the delineation of brain involvement of NF1 patients [10].

Hsieh HY et al. investigated the neurological complications and characteristics of intracranial lesions in patients with NF1 in Taiwan. They found that neurofibromatosis bright objects are frequent neuroimaging findings in patients with NF1, and are at high risk of transforming into tumors. The incidences of epilepsy and young-onset cerebral infarction in NF1 patients in this study are higher than those in the general population. Authors concluded that neuroimaging studies are essential for NF1 patients to determine the extent of neurological complications, although the imaging findings may not be completely correlated with the clinical manifestations [14].

The normal appearing cerebellar white matter in our child, when compared with the contralateral not affected hemisphere and with healthy ten controls, did not show nor ADC neither FA significant changes. On the contrary, at MRS we observed an increased Cho/Cr ratio (Figure 1C-G) which, despite not being specific to NF1 disease, may be related to increased membrane turnover [13]. In other words, in our NF1 affected child, a possible correlation between DWI and MRS was not documented. In fact, in the normal appearing cerebellar white matter we found normal ADC and FA value while at MRS a Cho/Cr ratio increase was demonstrated (Figure 1C-G). Nevertheless, being not specific of NF1 patients we can add nothing about the possible predictive value of Cho/Cr ratio increase in normal appearing white matter. Anyway, it should be stressed that this is just a family report and MRS findings here reported need to be confirmed in a large NF1 paediatric sample.

In addition, we would like to spend few sentences on the pontine hamartoma found out in our child and the differential diagnosis of brainstem lesions in paediatric age. The advent of MR imaging has simplified the diagnosis of brainstem tumors and it has also given new information that has allowed the identification of subgroups with different therapeutic options and prognoses [4,5]. As above reported, optic pathway [4] and brainstem [5] gliomas are the prevalent CNS tumours in NF1. The pons is the most common location of tumors originating in the brainstem in paediatric age, whose prognosis for most patients is definitely poor. On the contrary, in NF1 affected children, an isolated focal pontine tumor localization represents an exceptional finding [4,5] and brainstem tumors show generally a very much better prognosis. In fact, it should be stressed that the brainstem tumors associated with NF1 are currently considered a subgroup with further separations into focal and diffuse tumors. Brainstem tumors are detected in about 9–10 % of patients with NF1 and medulla localization represents the most common site (70-82%) while there is a marked pontine predominance in subjects without NF1 [4]. Although brainstem tumors of NF1 show an identical imaging appearance to those that occur outside the spectrum of NF1, their clinical behavior is often strikingly different and lesion may show a clear slow tumor growth. Even in patients with tumors that have imaging appearances identical to diffuse pontine tumors often have relatively indolent clinical course. Our patient, in fact, after four-year of follow-up showed both unchanged clinical pictures and unchanged size of pontine hamartoma. Most T2 hyperintensities regress with age and seem to be benign, however, children with a large number and volume of T2 hyperintensities should be followed closely with regular neuroimaging examinations because of an increased risk of proliferative change. T2 hyperintensities occur commonly in children with NF1 and are most prevalent between the ages of 4 and 10. Paul D. Griffiths et al. have shown a high frequency of brain tumors in children with NF1 and demonstrated proliferative change T2 hyperintensities in 11% of 46 children; they described 8 cases with brain tumors, 6 of which seemed to have developed after the follow-up imaging study. Five tumors were transformed from T2 hyperintensities, and 1 tumor that was not overlapped in position with a previous T2 hyperintensities [15]. Recent works have established that T2 hyperintensities do not have developmental potential, although changes in FA despite the disappearance or reductions of T2 hyperintensities have been confirmed due to DTI, supporting thus the hypothesis that microstructural damage occurs in specific brain regions of NF1 patients [16,17]. We found a single lesion in our patient and even if from literature data seems that the developmental potential is to be reserved in case of multiple lesions, we followed closely our patient, with regular neuroimaging examinations, essential for NF1 patients to detect any change in the extent of neurological complications, not completely correlated with the clinical manifestations.

Finally, we believe that there are at least some good reasons that make this family so interesting to be described. Firstly, four years of follow-up allowed us to confirm both the well known slow growth of these lesions (Figure 2: 2007 and 2010) and the peculiar focal pontine localization. Secondly, comparing father and son, the well known intra-familial phenotype variability, even at the MRI, MRS and DWI investigations levels, was confirmed.

## Consent

Written informed consent was obtained from the patient’s parents for publication of this Case report and any accompanying images. A copy of the written consent is available for review by the Editor-in-Chief of this journal.

## Competing interest

We have no competing interest in publishing this paper.

## Authors’ contributions

PP: Conceptualized and designed the study. He took care of the Family during the 4-year-follow-up, drafted the initial manuscript and approved the final manuscript as submitted. SP: He took care of the Family during the 4-year-follow-up outpatient service and approved the final manuscript as submitted. MCP: She took care of the Family during the 4-year-follow-up outpatient service and approved the final manuscript as submitted. FN: He took care of the Family during the 4-year-follow-up outpatient service and approved the final manuscript as submitted. IT: She performed the genetic analysis of the Family and approved the final manuscript as submitted. AB: He performed the neuroradiological investigations (structural and functional) of the Family and approved the final manuscript as submitted. MPV: She took care of the Family during the 4-year-follow-up, drafted the initial manuscript and approved the final manuscript as submitted. All authors read and approved the final manuscript.
